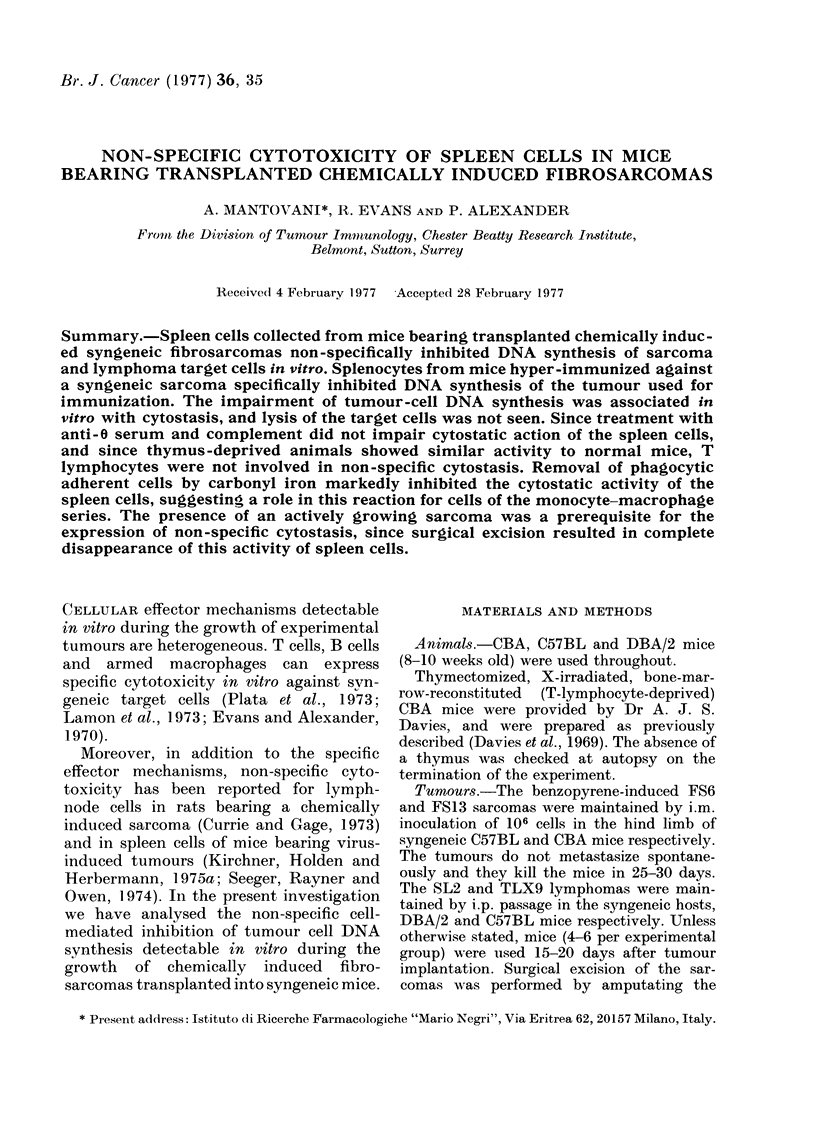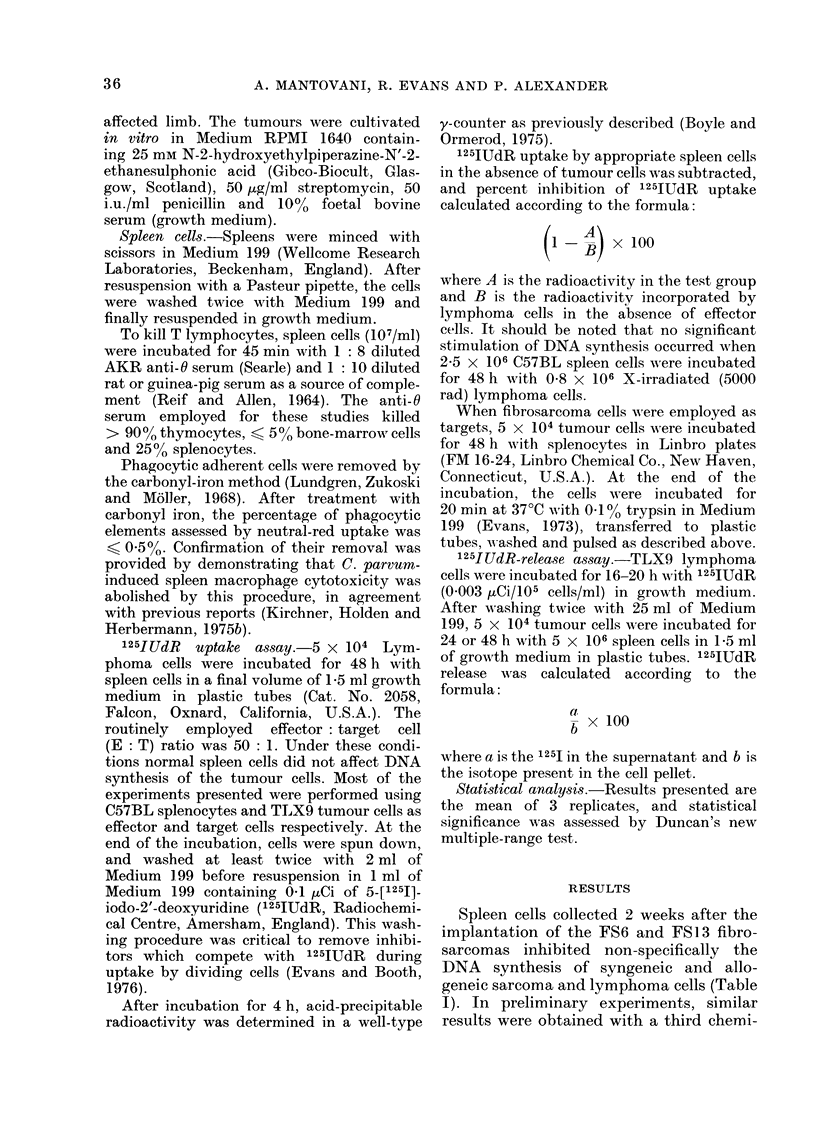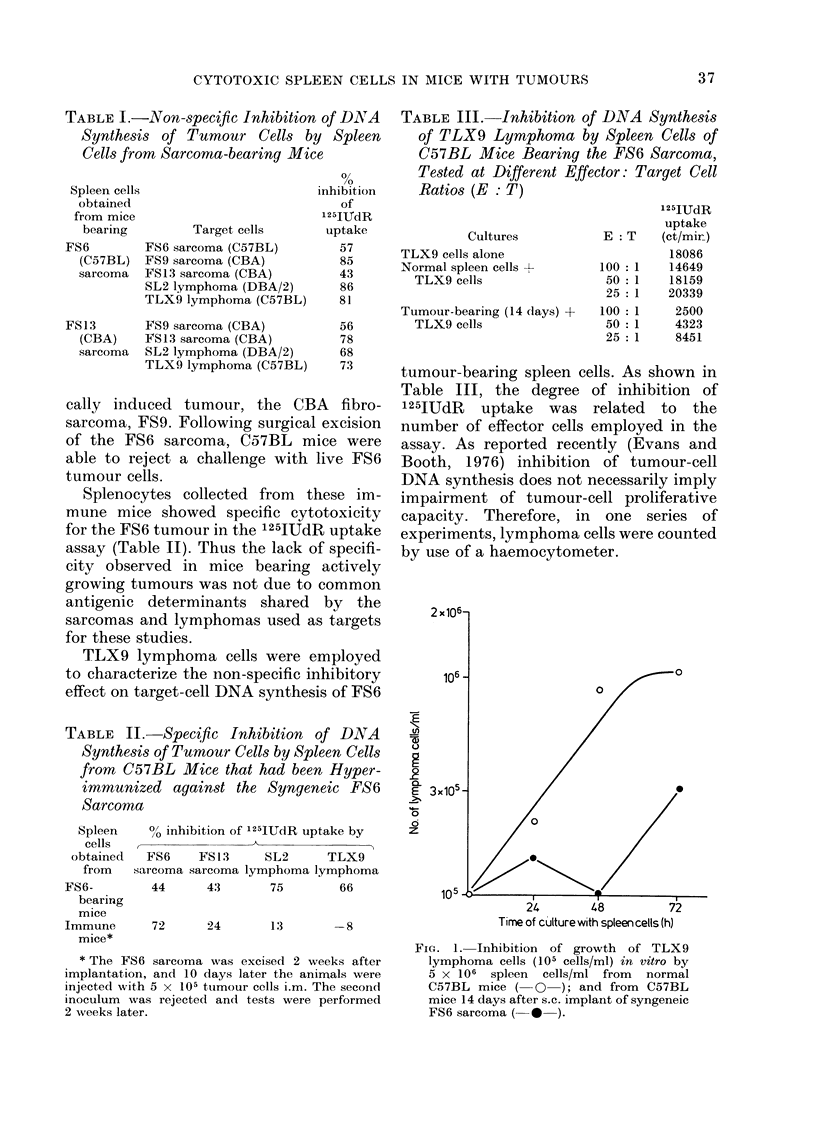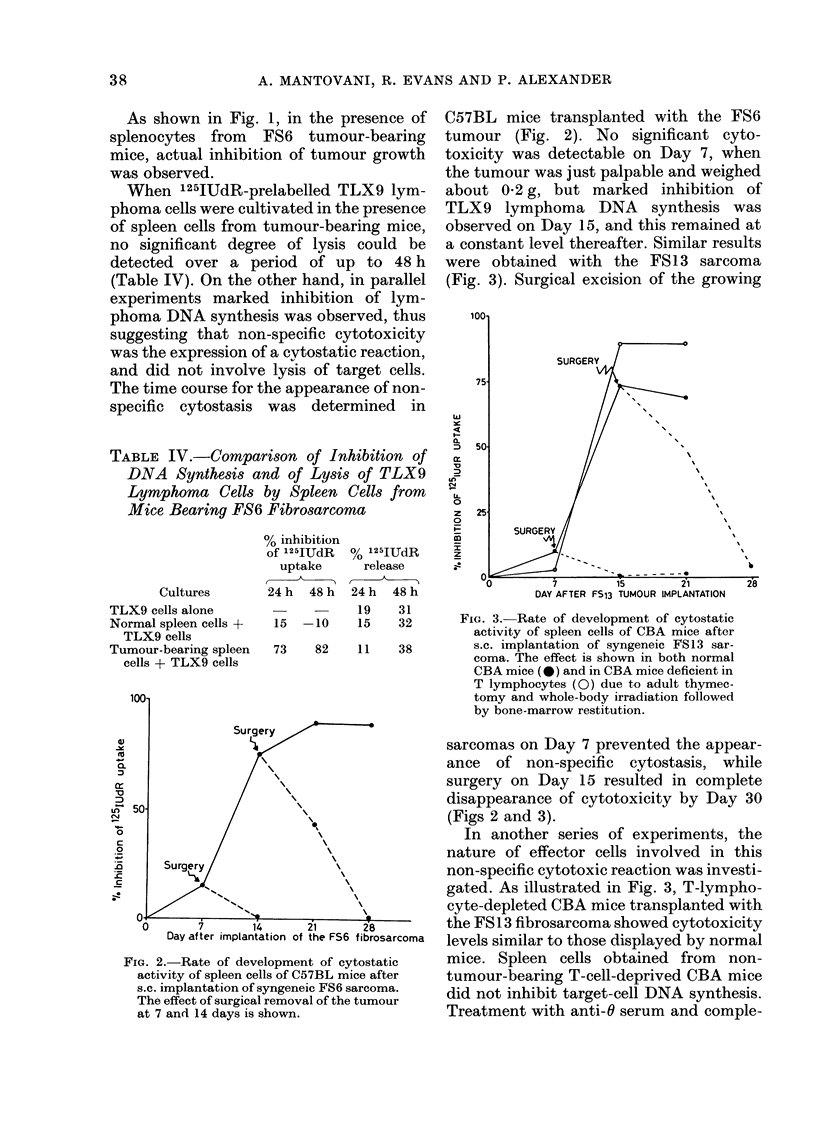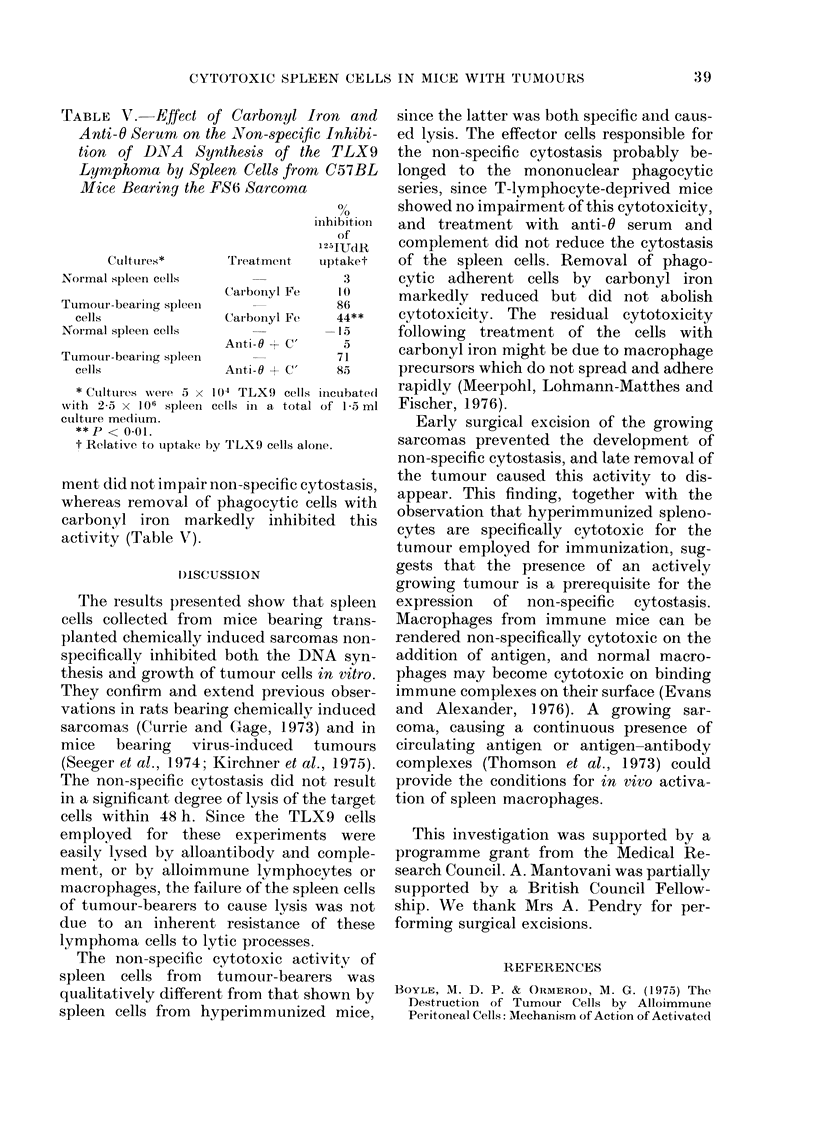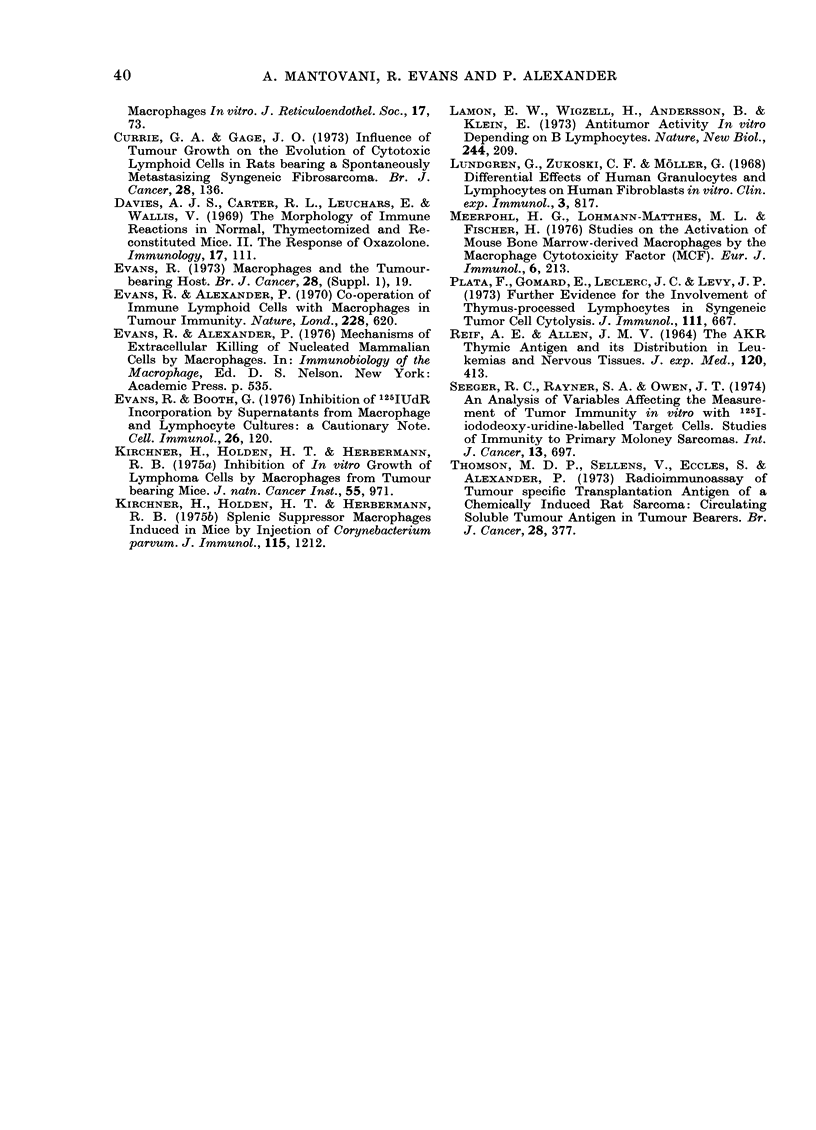# Non-specific cytotoxicity of spleen cells in mice bearing transplanted chemically induced fibrosarcomas.

**DOI:** 10.1038/bjc.1977.151

**Published:** 1977-07

**Authors:** A. Mantovani, R. Evans, P. Alexander

## Abstract

Spleen cells collected from mice bearing transplanted chemically induced syngeneic fibrosarcomas non-specifically inhibited DNA synthesis of sarcoma and lymphoma target cells in vitro. Splenocytes from mice hyper-immunized against a syngeneic sarcoma specifically inhibited DNA synthesis of the tumour used for immunization. The impairment of tumour-cell DNA synthesis was associated in vitro with cytostasis, and lysis of the target cells was not seen. Since treatment with anti-theta serum and complement did not impair cytostatic action of the spleen cells, and since thymus-deprived animals showed similar activity to normal mice, T lymphocytes were not involved in non-specific cytostasis. Removal of phagocytic adherent cells by carbonyl iron markedly inhibited the cytostatic activity of the spleen cells, suggesting a role in this reaction for cells of the monocyte-macrophage series. The presence of an actively growing sarcoma was a prerequisite for the expression of non-specific cytostasis, since surgical excision resulted in complete disappearance of this activity of spleen cells.


*Br. J. Cancer* (1977) **36**, 35

### NON-SPECIFIC CYTOTOXICITY OF SPLEEN CELLS IN MICE BEARING TRANSPLANTED CHEMICALLY INDUCED FIBROSARCOMAS

A. MANTOVANI*, R. EVANS AND P. ALEXANDER

*From the Division of Tumour Immunology, Chester Beatty Research Institute, Belmont, Sutton, Surrey*

Received 4 February 1977 Accepted 28 February 1977

**Summary.—Spleen cells collected from mice bearing transplanted chemically induced syngeneic fibrosarcomas non-specifically inhibited DNA synthesis of sarcoma and lymphoma target cells *in vitro*. Splenocytes from mice hyper-immunized against a syngeneic sarcoma specifically inhibited DNA synthesis of the tumour used for immunization. The impairment of tumour-cell DNA synthesis was associated *in vitro* with cytostasis, and lysis of the target cells was not seen. Since treatment with anti-θ serum and complement did not impair cytostatic action of the spleen cells, and since thymus-deprived animals showed similar activity to normal mice, T lymphocytes were not involved in non-specific cytostasis. Removal of phagocytic adherent cells by carbonyl iron markedly inhibited the cytostatic activity of the spleen cells, suggesting a role in this reaction for cells of the monocyte–macrophage series. The presence of an actively growing sarcoma was a prerequisite for the expression of non-specific cytostasis, since surgical excision resulted in complete disappearance of this activity of spleen cells.**

CELLULAR effector mechanisms detectable *in vitro* during the growth of experimental tumours are heterogeneous. T cells, B cells and armed macrophages can express specific cytotoxicity *in vitro* against syngeneic target cells (Plata *et al.*, 1973; Lamon *et al.*, 1973; Evans and Alexander, 1970).

Moreover, in addition to the specific effector mechanisms, non-specific cytotoxicity has been reported for lymph-node cells in rats bearing a chemically induced sarcoma (Currie and Gage, 1973) and in spleen cells of mice bearing virus-induced tumours (Kirchner, Holden and Herbermann, 1975*a*; Seeger, Rayner and Owen, 1974). In the present investigation we have analysed the non-specific cell-mediated inhibition of tumour cell DNA synthesis detectable *in vitro* during the growth of chemically induced fibrosarcomas transplanted into syngeneic mice.

### MATERIALS AND METHODS

*Animals.*—CBA, C57BL and DBA/2 mice (8–10 weeks old) were used throughout.

Thymectomized, X-irradiated, bone-marrow-reconstituted (T-lymphocyte-deprived) CBA mice were provided by Dr A. J. S. Davies, and were prepared as previously described (Davies *et al.*, 1969). The absence of a thymus was checked at autopsy on the termination of the experiment.

*Tumours.*—The benzopyrene-induced FS6 and FS13 sarcomas were maintained by i.m. inoculation of $10^6$ cells in the hind limb of syngeneic C57BL and CBA mice respectively. The tumours do not metastasize spontaneously and they kill the mice in 25–30 days. The SL2 and TLX9 lymphomas were maintained by i.p. passage in the syngeneic hosts, DBA/2 and C57BL mice respectively. Unless otherwise stated, mice (4–6 per experimental group) were used 15–20 days after tumour implantation. Surgical excision of the sarcomas was performed by amputating the

* Present address: Istituto di Ricerche Farmacologiche "Mario Negri", Via Eritrea 62, 20157 Milano, Italy.

affected limb. The tumours were cultivated *in vitro* in Medium RPMI 1640 containing 25 mM N-2-hydroxyethylpiperazine-N′-2-ethanesulphonic acid (Gibco-Biocult, Glasgow, Scotland), 50 μg/ml streptomycin, 50 i.u./ml penicillin and 10% foetal bovine serum (growth medium).

*Spleen cells*.—Spleens were minced with scissors in Medium 199 (Wellcome Research Laboratories, Beckenham, England). After resuspension with a Pasteur pipette, the cells were washed twice with Medium 199 and finally resuspended in growth medium.

To kill T lymphocytes, spleen cells ($10^7$/ml) were incubated for 45 min with 1 : 8 diluted AKR anti-θ serum (Searle) and 1 : 10 diluted rat or guinea-pig serum as a source of complement (Reif and Allen, 1964). The anti-θ serum employed for these studies killed $> 90\%$ thymocytes, $\leqslant 5\%$ bone-marrow cells and 25% splenocytes.

Phagocytic adherent cells were removed by the carbonyl-iron method (Lundgren, Zukoski and Möller, 1968). After treatment with carbonyl iron, the percentage of phagocytic elements assessed by neutral-red uptake was $\leqslant 0{\cdot}5\%$. Confirmation of their removal was provided by demonstrating that *C. parvum*-induced spleen macrophage cytotoxicity was abolished by this procedure, in agreement with previous reports (Kirchner, Holden and Herbermann, 1975*b*).

*$^{125}$IUdR uptake assay*.—$5 \times 10^4$ Lymphoma cells were incubated for 48 h with spleen cells in a final volume of 1·5 ml growth medium in plastic tubes (Cat. No. 2058, Falcon, Oxnard, California, U.S.A.). The routinely employed effector : target cell (E : T) ratio was 50 : 1. Under these conditions normal spleen cells did not affect DNA synthesis of the tumour cells. Most of the experiments presented were performed using C57BL splenocytes and TLX9 tumour cells as effector and target cells respectively. At the end of the incubation, cells were spun down, and washed at least twice with 2 ml of Medium 199 before resuspension in 1 ml of Medium 199 containing 0·1 μCi of 5-[$^{125}$I]-iodo-2′-deoxyuridine ($^{125}$IUdR, Radiochemical Centre, Amersham, England). This washing procedure was critical to remove inhibitors which compete with $^{125}$IUdR during uptake by dividing cells (Evans and Booth, 1976).

After incubation for 4 h, acid-precipitable radioactivity was determined in a well-type γ-counter as previously described (Boyle and Ormerod, 1975).

$^{125}$IUdR uptake by appropriate spleen cells in the absence of tumour cells was subtracted, and percent inhibition of $^{125}$IUdR uptake calculated according to the formula:

$$\left(1 - \frac{A}{B}\right) \times 100$$

where $A$ is the radioactivity in the test group and $B$ is the radioactivity incorporated by lymphoma cells in the absence of effector cells. It should be noted that no significant stimulation of DNA synthesis occurred when $2{\cdot}5 \times 10^6$ C57BL spleen cells were incubated for 48 h with $0{\cdot}8 \times 10^6$ X-irradiated (5000 rad) lymphoma cells.

When fibrosarcoma cells were employed as targets, $5 \times 10^4$ tumour cells were incubated for 48 h with splenocytes in Linbro plates (FM 16-24, Linbro Chemical Co., New Haven, Connecticut, U.S.A.). At the end of the incubation, the cells were incubated for 20 min at 37°C with 0·1% trypsin in Medium 199 (Evans, 1973), transferred to plastic tubes, washed and pulsed as described above.

*$^{125}$IUdR-release assay*.—TLX9 lymphoma cells were incubated for 16–20 h with $^{125}$IUdR (0·003 μCi/$10^5$ cells/ml) in growth medium. After washing twice with 25 ml of Medium 199, $5 \times 10^4$ tumour cells were incubated for 24 or 48 h with $5 \times 10^6$ spleen cells in 1·5 ml of growth medium in plastic tubes. $^{125}$IUdR release was calculated according to the formula:

$$\frac{a}{b} \times 100$$

where $a$ is the $^{125}$I in the supernatant and $b$ is the isotope present in the cell pellet.

*Statistical analysis*.—Results presented are the mean of 3 replicates, and statistical significance was assessed by Duncan's new multiple-range test.

### RESULTS

Spleen cells collected 2 weeks after the implantation of the FS6 and FS13 fibrosarcomas inhibited non-specifically the DNA synthesis of syngeneic and allogeneic sarcoma and lymphoma cells (Table I). In preliminary experiments, similar results were obtained with a third chemi-

TABLE I.—*Non-specific Inhibition of DNA Synthesis of Tumour Cells by Spleen Cells from Sarcoma-bearing Mice*

| Spleen cells obtained from mice bearing | Target cells | % inhibition of $^{125}$IUdR uptake |
|---|---|---|
| FS6 (C57BL) sarcoma | FS6 sarcoma (C57BL) | 57 |
| | FS9 sarcoma (CBA) | 85 |
| | FS13 sarcoma (CBA) | 43 |
| | SL2 lymphoma (DBA/2) | 86 |
| | TLX9 lymphoma (C57BL) | 81 |
| FS13 (CBA) sarcoma | FS9 sarcoma (CBA) | 56 |
| | FS13 sarcoma (CBA) | 78 |
| | SL2 lymphoma (DBA/2) | 68 |
| | TLX9 lymphoma (C57BL) | 73 |

cally induced tumour, the CBA fibrosarcoma, FS9. Following surgical excision of the FS6 sarcoma, C57BL mice were able to reject a challenge with live FS6 tumour cells.

Splenocytes collected from these immune mice showed specific cytotoxicity for the FS6 tumour in the $^{125}$IUdR uptake assay (Table II). Thus the lack of specificity observed in mice bearing actively growing tumours was not due to common antigenic determinants shared by the sarcomas and lymphomas used as targets for these studies.

TLX9 lymphoma cells were employed to characterize the non-specific inhibitory effect on target-cell DNA synthesis of FS6

TABLE II.—*Specific Inhibition of DNA Synthesis of Tumour Cells by Spleen Cells from C57BL Mice that had been Hyperimmunized against the Syngeneic FS6 Sarcoma*

| Spleen cells obtained from | % inhibition of $^{125}$IUdR uptake by FS6 sarcoma | FS13 sarcoma | SL2 lymphoma | TLX9 lymphoma |
|---|---|---|---|---|
| FS6-bearing mice | 44 | 43 | 75 | 66 |
| Immune mice* | 72 | 24 | 13 | −8 |

* The FS6 sarcoma was excised 2 weeks after implantation, and 10 days later the animals were injected with $5 \times 10^5$ tumour cells i.m. The second inoculum was rejected and tests were performed 2 weeks later.

TABLE III.—*Inhibition of DNA Synthesis of TLX9 Lymphoma by Spleen Cells of C57BL Mice Bearing the FS6 Sarcoma, Tested at Different Effector: Target Cell Ratios (E : T)*

| Cultures | E : T | $^{125}$IUdR uptake (ct/min) |
|---|---|---|
| TLX9 cells alone | | 18086 |
| Normal spleen cells + TLX9 cells | 100 : 1 | 14649 |
| | 50 : 1 | 18159 |
| | 25 : 1 | 20339 |
| Tumour-bearing (14 days) + TLX9 cells | 100 : 1 | 2500 |
| | 50 : 1 | 4323 |
| | 25 : 1 | 8451 |

tumour-bearing spleen cells. As shown in Table III, the degree of inhibition of $^{125}$IUdR uptake was related to the number of effector cells employed in the assay. As reported recently (Evans and Booth, 1976) inhibition of tumour-cell DNA synthesis does not necessarily imply impairment of tumour-cell proliferative capacity. Therefore, in one series of experiments, lymphoma cells were counted by use of a haemocytometer.

FIG. 1.—Inhibition of growth of TLX9 lymphoma cells ($10^5$ cells/ml) *in vitro* by $5 \times 10^6$ spleen cells/ml from normal C57BL mice (—○—); and from C57BL mice 14 days after s.c. implant of syngeneic FS6 sarcoma (—●—).

As shown in Fig. 1, in the presence of splenocytes from FS6 tumour-bearing mice, actual inhibition of tumour growth was observed.

When $^{125}$IUdR-prelabelled TLX9 lymphoma cells were cultivated in the presence of spleen cells from tumour-bearing mice, no significant degree of lysis could be detected over a period of up to 48 h (Table IV). On the other hand, in parallel experiments marked inhibition of lymphoma DNA synthesis was observed, thus suggesting that non-specific cytotoxicity was the expression of a cytostatic reaction, and did not involve lysis of target cells. The time course for the appearance of non-specific cytostasis was determined in C57BL mice transplanted with the FS6 tumour (Fig. 2). No significant cytotoxicity was detectable on Day 7, when the tumour was just palpable and weighed about 0·2 g, but marked inhibition of TLX9 lymphoma DNA synthesis was observed on Day 15, and this remained at a constant level thereafter. Similar results were obtained with the FS13 sarcoma (Fig. 3). Surgical excision of the growing sarcomas on Day 7 prevented the appearance of non-specific cytostasis, while surgery on Day 15 resulted in complete disappearance of cytotoxicity by Day 30 (Figs 2 and 3).

TABLE IV.—*Comparison of Inhibition of DNA Synthesis and of Lysis of TLX9 Lymphoma Cells by Spleen Cells from Mice Bearing FS6 Fibrosarcoma*

| Cultures | % inhibition of $^{125}$IUdR uptake | | % $^{125}$IUdR release | |
|---|---|---|---|---|
| | 24 h | 48 h | 24 h | 48 h |
| TLX9 cells alone | — | — | 19 | 31 |
| Normal spleen cells + TLX9 cells | 15 | −10 | 15 | 32 |
| Tumour-bearing spleen cells + TLX9 cells | 73 | 82 | 11 | 38 |

FIG. 2.—Rate of development of cytostatic activity of spleen cells of C57BL mice after s.c. implantation of syngeneic FS6 sarcoma. The effect of surgical removal of the tumour at 7 and 14 days is shown.

FIG. 3.—Rate of development of cytostatic activity of spleen cells of CBA mice after s.c. implantation of syngeneic FS13 sarcoma. The effect is shown in both normal CBA mice (●) and in CBA mice deficient in T lymphocytes (○) due to adult thymectomy and whole-body irradiation followed by bone-marrow restitution.

In another series of experiments, the nature of effector cells involved in this non-specific cytotoxic reaction was investigated. As illustrated in Fig. 3, T-lymphocyte-depleted CBA mice transplanted with the FS13 fibrosarcoma showed cytotoxicity levels similar to those displayed by normal mice. Spleen cells obtained from non-tumour-bearing T-cell-deprived CBA mice did not inhibit target-cell DNA synthesis. Treatment with anti-$\theta$ serum and comple-

TABLE V.—*Effect of Carbonyl Iron and Anti-θ Serum on the Non-specific Inhibition of DNA Synthesis of the TLX9 Lymphoma by Spleen Cells from C57BL Mice Bearing the FS6 Sarcoma*

| Cultures* | Treatment | % inhibition of $^{125}$IUdR uptake† |
|---|---|---|
| Normal spleen cells | — | 3 |
| | Carbonyl Fe | 10 |
| Tumour-bearing spleen | — | 86 |
| cells | Carbonyl Fe | 44** |
| Normal spleen cells | — | −15 |
| | Anti-θ + C′ | 5 |
| Tumour-bearing spleen | — | 71 |
| cells | Anti-θ + C′ | 85 |

* Cultures were $5 \times 10^4$ TLX9 cells incubated with $2 \cdot 5 \times 10^6$ spleen cells in a total of 1·5 ml culture medium.

** $P < 0 \cdot 01$.

† Relative to uptake by TLX9 cells alone.

ment did not impair non-specific cytostasis, whereas removal of phagocytic cells with carbonyl iron markedly inhibited this activity (Table V).

### DISCUSSION

The results presented show that spleen cells collected from mice bearing transplanted chemically induced sarcomas non-specifically inhibited both the DNA synthesis and growth of tumour cells *in vitro*. They confirm and extend previous observations in rats bearing chemically induced sarcomas (Currie and Gage, 1973) and in mice bearing virus-induced tumours (Seeger *et al.*, 1974; Kirchner *et al.*, 1975). The non-specific cytostasis did not result in a significant degree of lysis of the target cells within 48 h. Since the TLX9 cells employed for these experiments were easily lysed by alloantibody and complement, or by alloimmune lymphocytes or macrophages, the failure of the spleen cells of tumour-bearers to cause lysis was not due to an inherent resistance of these lymphoma cells to lytic processes.

The non-specific cytotoxic activity of spleen cells from tumour-bearers was qualitatively different from that shown by spleen cells from hyperimmunized mice, since the latter was both specific and caused lysis. The effector cells responsible for the non-specific cytostasis probably belonged to the mononuclear phagocytic series, since T-lymphocyte-deprived mice showed no impairment of this cytotoxicity, and treatment with anti-θ serum and complement did not reduce the cytostasis of the spleen cells. Removal of phagocytic adherent cells by carbonyl iron markedly reduced but did not abolish cytotoxicity. The residual cytotoxicity following treatment of the cells with carbonyl iron might be due to macrophage precursors which do not spread and adhere rapidly (Meerpohl, Lohmann-Matthes and Fischer, 1976).

Early surgical excision of the growing sarcomas prevented the development of non-specific cytostasis, and late removal of the tumour caused this activity to disappear. This finding, together with the observation that hyperimmunized splenocytes are specifically cytotoxic for the tumour employed for immunization, suggests that the presence of an actively growing tumour is a prerequisite for the expression of non-specific cytostasis. Macrophages from immune mice can be rendered non-specifically cytotoxic on the addition of antigen, and normal macrophages may become cytotoxic on binding immune complexes on their surface (Evans and Alexander, 1976). A growing sarcoma, causing a continuous presence of circulating antigen or antigen–antibody complexes (Thomson *et al.*, 1973) could provide the conditions for *in vivo* activation of spleen macrophages.

This investigation was supported by a programme grant from the Medical Research Council. A. Mantovani was partially supported by a British Council Fellowship. We thank Mrs A. Pendry for performing surgical excisions.

### REFERENCES

BOYLE, M. D. P. & ORMEROD, M. G. (1975) The Destruction of Tumour Cells by Alloimmune Peritoneal Cells: Mechanism of Action of Activated

Macrophages *In vitro*. *J. Reticuloendothel. Soc.*, **17**, 73.

CURRIE, G. A. & GAGE, J. O. (1973) Influence of Tumour Growth on the Evolution of Cytotoxic Lymphoid Cells in Rats bearing a Spontaneously Metastasizing Syngeneic Fibrosarcoma. *Br. J. Cancer*, **28**, 136.

DAVIES, A. J. S., CARTER, R. L., LEUCHARS, E. & WALLIS, V. (1969) The Morphology of Immune Reactions in Normal, Thymectomized and Reconstituted Mice. II. The Response of Oxazolone. *Immunology*, **17**, 111.

EVANS, R. (1973) Macrophages and the Tumour-bearing Host. *Br. J. Cancer*, **28**, (Suppl. 1), 19.

EVANS, R. & ALEXANDER, P. (1970) Co-operation of Immune Lymphoid Cells with Macrophages in Tumour Immunity. *Nature, Lond.*, **228**, 620.

EVANS, R. & ALEXANDER, P. (1976) Mechanisms of Extracellular Killing of Nucleated Mammalian Cells by Macrophages. In: *Immunobiology of the Macrophage*, Ed. D. S. Nelson. New York: Academic Press. p. 535.

EVANS, R. & BOOTH, G. (1976) Inhibition of $^{125}$IUdR Incorporation by Supernatants from Macrophage and Lymphocyte Cultures: a Cautionary Note. *Cell. Immunol.*, **26**, 120.

KIRCHNER, H., HOLDEN, H. T. & HERBERMANN, R. B. (1975*a*) Inhibition of *In vitro* Growth of Lymphoma Cells by Macrophages from Tumour bearing Mice. *J. natn. Cancer Inst.*, **55**, 971.

KIRCHNER, H., HOLDEN, H. T. & HERBERMANN, R. B. (1975*b*) Splenic Suppressor Macrophages Induced in Mice by Injection of *Corynebacterium parvum*. *J. Immunol.*, **115**, 1212.

LAMON, E. W., WIGZELL, H., ANDERSSON, B. & KLEIN, E. (1973) Antitumor Activity *In vitro* Depending on B Lymphocytes. *Nature, New Biol.*, **244**, 209.

LUNDGREN, G., ZUKOSKI, C. F. & MÖLLER, G. (1968) Differential Effects of Human Granulocytes and Lymphocytes on Human Fibroblasts *in vitro*. *Clin. exp. Immunol.*, **3**, 817.

MEERPOHL, H. G., LOHMANN-MATTHES, M. L. & FISCHER, H. (1976) Studies on the Activation of Mouse Bone Marrow-derived Macrophages by the Macrophage Cytotoxicity Factor (MCF). *Eur. J. Immunol.*, **6**, 213.

PLATA, F., GOMARD, E., LECLERC, J. C. & LEVY, J. P. (1973) Further Evidence for the Involvement of Thymus-processed Lymphocytes in Syngeneic Tumor Cell Cytolysis. *J. Immunol.*, **111**, 667.

REIF, A. E. & ALLEN, J. M. V. (1964) The AKR Thymic Antigen and its Distribution in Leukemias and Nervous Tissues. *J. exp. Med.*, **120**, 413.

SEEGER, R. C., RAYNER, S. A. & OWEN, J. T. (1974) An Analysis of Variables Affecting the Measurement of Tumor Immunity *in vitro* with $^{125}$I-iododeoxy-uridine-labelled Target Cells. Studies of Immunity to Primary Moloney Sarcomas. *Int. J. Cancer*, **13**, 697.

THOMSON, M. D. P., SELLENS, V., ECCLES, S. & ALEXANDER, P. (1973) Radioimmunoassay of Tumour specific Transplantation Antigen of a Chemically Induced Rat Sarcoma: Circulating Soluble Tumour Antigen in Tumour Bearers. *Br. J. Cancer*, **28**, 377.